# The Optimal Digestible Leucine-to-Lysine Ratio in Wheat-Based Diets for Finishing Broilers

**DOI:** 10.3390/ani15142092

**Published:** 2025-07-15

**Authors:** Diana Siebert, Christian Scharch

**Affiliations:** 1 CJ Europe GmbH, 60549 Frankfurt am Main, Germany; 2 Feedtest, 06193 Wettin-Löbejün, Germany; scharch@feedtest.de

**Keywords:** leucine, recommendations, finisher broiler, leucine-to-lysine ratio

## Abstract

Male Ross 308 finisher (19 to 33 days) broiler chickens (*n* = 672) were used to determine the standardized ileal digestible (SID) leucine (Leu)-to-lysine (Lys) ratio for the maximum growth performance and slaughter parameters. A basal diet (incl. wheat, triticale, soybean meal, sunflower meal, and synthetic amino acids) deficient in Leu was formulated, and supplementary L-Leu was used to achieve 0.9 and 1.25 SID Leu-to-Lys ratios in increments of 0.05. A slight deficiency in lysine was implemented to truly relate broiler performance to changes in the SID Leu-to-Lys ratio. Diets were fed to broilers in seven pens (12 broilers each). Eight birds per pen were used for carcass evaluation on day 33. Data analysis indicated that SID Leu-to-Lys ratios between 0.96 and 1.025 were required to achieve 95% and 98% of the maximum body weight gain, daily feed consumption, and the European production efficacy factor, respectively. The estimation of recommended SID Leu-to-Lys ratios for the feed conversion ratio and carcass parameters was not possible.

## Simple Summary

In wheat-based diets, the leucine content can be low, especially in a low-crude-protein diet. A low leucine content may limit the maximum performance and thus the efficiency of broiler chickens. The addition of crystalline L-leucine is a possible solution to a low-protein wheat-based diet. However, little is known about the ideal leucine-to-lysine ratio in finishing broilers. Thus, the aim of this study was to determine the optimal ratio to maximize growth performance and slaughter parameters. In a trial facility, 672 male Ross 308 broilers were reared with a commercial starter and grower feed followed by allocation to one of eight dietary treatments with consecutive increasing amounts of L-leucine. The supplementation of L-leucine with a leucine-deficient basal diet led to an increase in the growth and feed intake of finishing broilers. A data analysis of body weight gain, daily feed consumption, and the European production efficacy factor indicated that leucine-to-lysine ratios between 0.96 and 1.025 are required to achieve 95% and 98% of the maximum performance, respectively.

## 1. Introduction

One of the major challenges of the livestock industry, in particular the broiler industry, is to meet the growing demand for food without compromising environmental integrity. Previous studies demonstrated that feed is a major source of CO_2_ emission [1,2], and consequently, the reformulation of feed can be an effective tool for reducing the environmental impact. Amongst others, reducing the dietary crude protein (CP) content in broiler diets has been associated with decreased nitrogen excretion and increased nitrogen-retention efficiency. In a 48-day rearing trial, the nitrogen excretion of male broilers was reduced by 9.5% and 17% when the dietary CP level was reduced by 1.5% and 3%, respectively [3]. Over two experiments with growing–finishing male broilers and consecutive reduced CP levels but a stable amino acid (AA) content, more recent research confirmed that the nitrogen-retention efficiency increased with decreasing CP content (+3.5 points per CP percentage point) [4]. Furthermore, low-CP diets have the ability to decrease the incidence of pododermatites [5], which is an important health and welfare indicator.

Despite the fact that reduced dietary CP is connected to reduced nitrogen excretion and litter content, and consequently decreased eutrophication, there may be a reduced growth performance of the birds. Some studies failed to demonstrate comparable growth in low-CP diets to that of high-protein diets [6,7]. A recent study showed no significant reduction in body weight or the FCR when birds were fed with an up to 3%-point CP reduction with a stable AA content accompanied by a significant reduction in the nitrogen content in the excreta [8]. Low-CP diets without carefully considered essential AAs certainly will lead to a reduction in performance [7]. One prerequisite of such a consideration is the knowledge of the broiler’s needs for each essential AA.

Leucine (Leu), valine (Val), and isoleucine (Ile) form the group of branched-chain AAs (BCAAs). BCAA metabolism is strongly connected to protein synthesis and consequently growth performance. BCAAs are involved in lymphoid organ development and immune response, the modulation of the gut microbiome, and intestinal development [9,10]. On one hand, amongst all BCAAs, Leu is known to have the most potent stimulating effect on protein synthesis via the activation of the mechanistic target of the rapamycin (mTOR) pathway [9]. On the other hand, BCAAs share a common catabolism process [11]; consequently, interactions or competitive effects between BCAAs may influence their requirements. A study using Cobb 500 broiler chickens in a 2 by 3 by 3 factorial design (2 Leu levels (1.28% or 1.83%), 3 valine levels (0.65%, 0.90%, or 1.20%), and 3 isoleucine levels (0.54%, 0.79%, or 1.09%)) demonstrated that a high Leu level in feed decreases the growth performance of birds fed with diets marginal in Val or Ile content but restores their performance when Val and Ile were supplemented at adequate or high levels [12]. While an oversupply of Leu is common in corn–soybean-based formulations and therefore rather well investigated, research on the optimal Leu-to-lysine (Lys) ratio is lacking, in particular, in the finisher period. In comparison to wheat, corn is lower in tryptophan, arginine, and phenylalanine, which also needs to be adjusted in feed formulations along with a higher Leu content. Dietary Leu content might be limited in a wheat-based diet; thus knowledge about the optimal Leu-to-Lys ratio is necessary in order to formulate diets which meet the animal’s needs. The aim of this study was to determine the optimal Leu-to-Lys ratio in a dose–response trial in wheat-based diets in chickens during the finisher phase.

## 2. Materials and Methods

### 2.1. Animals and Housing

A feeding trial was performed with a total of 672 male Ross 308 broilers. The chickens were obtained at the day of hatch from a local German hatchery (Geflügelhof Möckern, 39291 Möckern, Germany). The birds were randomly assigned in groups of 14 chickens to experimental pens (one square meter) equipped with three nipple drinkers and a round feeder. Birds were reared commonly during the starter (0–10 days) and the grower phases (10–19 days) until the trial started at day 19. At day 19, the number of birds was reduced to 12 per pen by removing the lightest and the heaviest birds and the body weight was recorded individually. Birds were allocated to one of eight dietary treatments, while treatments were evenly spread across the pens. Feed and water were available ad libitum; feed consumption was recorded per pen. The bedding material consisted of wood shavings. Caked excreta patches around the drinkers were removed and fresh bedding material was added when required to prevent footpad lesions. Light and temperature regimes were maintained according to the breeder’s guidelines [13] and respective welfare legislations [14]. The period of darkness (night) increased from one hour at chick placement daily by one hour to achieve eight hours of darkness. Daily mortalities were recorded per pen throughout the rearing period (0–33 days). Birds were vaccinated against Newcastle disease and Gumboro at the age of 14 days.

### 2.2. Experimental Diets

Feed was produced at a specialized feed mill (Research Diet Services, Hoge Maat 10, 3961 NC Wijk bij Duurstede, The Netherlands). The starter and grower feed were formulated to meet the breeder’s recommendations [15]. The test diets (finisher phase) mainly consisted of wheat, triticale, soybean meal and sunflower meal, and supplementary amino acids, vitamins and minerals (Table 1). The dietary content of all essential amino acids, except Leu, were maintained constant among test diets. The diets were supplemented with commercially available phytase (Axtra^®^ Phy 20000 TPT2, Danisco Animal Nutrition, Marlborough, UK) and NSP-hydrolyzing enzymes (Ronozyme^®^ Multigrain, dsm-firmenich, Kaiseraugst, Switzerland) at recommended levels. No coccidiostat was included in the feeds. The digestible Lys content of the finisher diets was reduced about 10.8% as compared to the breeders recommendations of 1.06% [15] to truly relate the broiler performance to the Leu-to-Lys ratios. Sub-batches of the basal finisher diet were supplemented with the respective amounts of free L-Leu before pelleting to achieve the digestible Leu-to-Lys ratios between 0.9 and 1.25. The standardized ileal digestibility (SID) values of the raw materials are taken from published tables [16].

### 2.3. Diet Analysis

All diets were analyzed for their crude nutrients (proximate) and AA content according to the official methods of the VDLUFA [17]. The analyzed concentrations are shown in Table 2. In general, the calculated and analyzed nutrient values were in good agreement.

### 2.4. Performance Measurments and Carcass Evaluation

Birds were weighed (pen wise) at the start of this study (day 19) and on day 33, birds were weighed individually. In the same intervals, feed residues were determined. Feed offered was recorded continuously upon refilling the feeders. The weight of losses and culls was recorded upon occurrence. If possible, the reason of loss was determined (animal caretaker’s assessment).

Daily body weight gain per bird (BWG), average daily feed intake per bird (FI) and the feed conversion ratio (FCR) corrected for mortality were calculated. The European Efficacy Factor (EEF) was calculated as follows:

EEF = [(livability, % × BWG, kg)/(study duration in days × FCR)] × 100.

After the final weighing on day 33, eight birds per pen closest to pen mean BW were selected for carcass evaluation, identified by applying numbered wing tags and forwarded to a commercial slaughter facility for slaughter on the following morning. The cooled carcasses were separated into breast meat (without skin and bone, untrimmed), thighs and residual carcass on an individual bird basis. Wing tag number and weights of the cuts were recorded. Dressing as well as proportion of valuable cuts (based upon carcass and body weight, respectively) were calculated.

### 2.5. Statistical Analysis

The statistical unit for performance was ‘pen’ and for carcass evaluation ‘individual bird’. Prior to the statistical analysis, an outlier test (Grubb’s test) was conducted. There were no extreme or outlying data; consequently, no data were removed from the dataset. The performance data were analyzed using a one-factorial ANOVA with the fixed effect of treatment. Means were assessed by Tukey’s test to account for multiple comparisons. All statements of statistical significance were based on *p* < 0.05.

The statistical analysis of performance data was applied to the finisher period (i.e., 14 days of experimental feeding). Some parameters (body weight, BWG, FI, EEF) suggested an exponential dose–response relationship. A respective model was fitted to the data and recommended Leu-to-Lys ratios derived to achieve either 95 or 98% of the plateau performance.

$$\mathrm{The}\;\mathrm{exponential}\;\mathrm{model}:\;y=start+\left(asymptote-start_{E}\right)\times \left(1-e^{slope\times x}\right)$$

The exponential model was chosen as the data supported its application as well as the exponential model representing a physiological background. Observed data did not suggest the application of a broken-line or quadratic model. Other parameters such as the FCR and carcass evaluations did not allow for a regression approach to determine the recommended Leu-to-Lys ratios. The entire statistical analysis on the performance and carcass data was conducted using the software package SAS 9.4.

## 3. Results

### 3.1. Losses and Mortality

Due to increasing losses during the initial 10 days, antibiotic veterinary intervention was necessary. All animals were treated with Trimethoprim and Sulfamethoxazole via drinking water for a period of four consecutive days. After antibiotic intervention, the number of daily losses and culls decreased. During the finisher phase (d 19–33), only a few losses occurred (11 birds) over all treatments. Eight birds had cardiovascular failure, and one loss was due to a wing breakage. Treatment had no effect on mortality.

### 3.2. Growth Performance and European Efficacy Factor

At d 19, birds weighted on average 1011 g ± 6.89 g with no statistical differences between the treatments. At the end of the trial, the birds fed the 0.90% SID Leu-to-Lys ratio diet were lighter in comparison to those receiving diets with higher Leu-to-Lys ratios. The highest BW, BWG, FI, EEF, and the lowest FCR was realized in birds fed with the 1.20 SID Leu-to-Lys diet (Table 3).

### 3.3. Carcass Parameters

The birds fed the basal diet (0.90 SID Leu-to-Lys ratio) had the lowest proportion of valuable cuts. The carcass and breast meat yields in gram increased with increasing Leu-to-Lys ratios. The numerically highest carcass yield was reached with 1.20 SID Leu-to-Lys, while the total yield for breast meat and thighs peaked at 1.15 SID Leu-to-Lys. In relation to the carcass weight, the values remained comparable constant. The relative breast meat varies between 24.8% and 26.9% and the relative thigh weights vary between 29% and 30.6%, respectively (Table 4).

### 3.4. Estimation of SID Leu-to-Lys Requirement in Ross 308 Broilers

Table 5 summarizes the required SID Leu-to-Lys ratios to obtain 95% and 98% of plateau performance, respectively, as well as the respective plateau levels (including confidence limits). The estimates derived from BWG, FI and the EEF were similar, whilst the estimates based upon final BW are lower. Data analysis of BWG, FI and the EEF indicated that SID Leu-to-Lys ratios of 0.96 and 1.025 are needed to achieve 95% and 98% of maximum performance, respectively. Estimation of the recommended SID Leu-to-Lys for the FCR and carcass parameters was not possible.

## 4. Discussion

The aim of this study was to determine the SID Leu-to-Lys ratio in finishing broilers for optimal performance. To estimate optimal amino acid ratios a variety of statistical models can be applied, and the best fitting model also depends on the derived data. Usually, ratios estimated by the quadratic model tends to be overestimated [18], while linear models tend to underestimate the ratios [19]. Herein, the data did not suggest the application of a broken-line or a quadratic model, consequently the exponential model was chosen as best fitting option. Estimates were derived at 95% and 98% of maximum performance. Application of 98% of the plateau value can bring a higher safety in practical formulations, where Leu may not be critically considered because the optimal Leu-to-Lys ratio in finisher broilers are rarely investigated.

The BCAAs share a common metabolism, and especially excess Leu activates the degradation of Val and Ile [12,20]. The current study set the Ile-to-Lys ratio to 0.69 [15] and Val-to-Lys ratio to 0.80, following earlier research about the optimal Val-to-Lys ratio in broiler [21], and in agreement with the breeder’s recommendations of 0.78 [15]. Because these values are in good agreement with former research [21,22] and because the performance reached a plateau, but did not decrease with the higher Leu-to-Lys ratios, a relevant interaction effect between BCAAs seems to be unlikely up to a Leu-to-Lys ratio as high as 1.25.

The final BW of birds (Table 3) at SID Leu-to-Lys 0.90 (2290 g) exceeded what would have been expected from commercial performance objectives (2222 g at 33 days) [23]. About 40% of the final weight have been achieved already in the starter and grower phase in which the birds received a diet which was fulfilling all amino acid requirements. Nonetheless, the growth performance was markedly reduced when broilers were fed with a basal finisher diet deficient in Leu, proving a growth retarding effect of Leu deficiency. Furthermore, a clear dose–response relationship was observed and consequently an optimal SID Leu-to-Lys ratio for finishing broilers was estimated despite the comparable high overall performance. The breeder company recommend 1.10 as an optimal Leu-to-Lys ratio thru all growing stages [15]. In contrast, this study found lower recommendations, e.g., for BWG, an optimal Leu-to-Lys ratio of 0.962 and 1.025 to achieve 95% and 98% of maximum performance, respectively (Table 5). The optimal rearing conditions and small group size of birds may have contributed to these values. However, it can be also speculated, that the current breeder recommendations are overestimating the optimal Leu-to-Lys ratio in finisher birds between 19 and 33 days of age. The observation of this study is in good agreement with the findings of an earlier dose–response study in female Cobb 500 broilers [24]. Feeding the broilers during day 8–14 with one of seven experimental diets containing Leu-to-Lys ratios between 0.93 and 1.18, they estimated a SID Leu-to-Lys ratio of 1.07 and 1.06 for BWG with the curvilinear-plateau model and the quadratic regression analyses, respectively [24]. In a later trial with female Cobb 500 broilers from 8 to 21 days, Amirdhari et al. [25] confirmed an overall recommendation of 1.06 SID Leu-to-Lys in wheat-based feed formulas. Franco et al. [26] suggested a mean Leu-to-Lys ratio for performance parameters of 1.04 in female Cobb broilers from 8 to 17 days of age. However, estimates vary between 0.99 and 1.10 for different performance parameters and with varying statistical models. These variations were not observed at the current study. The estimated SID Leu-to-Lys ratios for different parameters are rather equal and vary between 0.930 and 0.962 and 0.994 and 1.025 to achieve 95% and 98% of the plateau, respectively. Comparable to this study, Franco et al. [26] was not able to estimate ratios for the FCR. Based on the results of a Leu dose–response trial in piglets (9–20 kg BW), the authors suggested that lighter pigs may require a higher Leu-to-Lys ratio to maximize their growth performance than heavier pigs [27]. Further research is necessary to confirm this hypothesis, and comparable data do not exist for broilers; however, it might offer a possible explanation why, especially in the current study with very high overall performance, rather low optimal Leu-to-Lys ratios were observed.

While several studies confirmed optimal SID Leu-to-Lys ratios lower than the breeder recommendations of 1.10 [15,24,26], a requirement study with Ross 308 contradicts the findings [28]. During the first 14 days, birds received seven experimental diets with a SID Lys level of 12.0 g per kg and SID Leu levels between 16.3 and 22.3 g per kg, corresponding to a Leu-to-Lys ratios between 1.36 and 1.86. They conclude that the requirement of Leu is between 18.67 g per kg (for BWG) and 19.85 g per kg (for breast meat yield), corresponding to an optimal Leu-to-Lys ratio of 1.56 and 1.65, respectively. The comparable higher requirements can be partially explained by the different methodologies used between Kratei and Shahir [28] and the current study (a requirement study versus a ratio study, respectively), which amongst others is characterized by the digestible Lys setting (an adequate Lys versus a sub-limiting Lys amount, respectively). Additionally, Kratei and Shahir [28] used a corn–soybean meal-based diet, leading to a SID Leu-to-Lys ratio in the basal diet of 1.36, which is already above the breeder’s recommendations of 1.10 [15] and exceeding the highest ratio of this study (Leu-to-Lys at 1.25). Because the total Leu content of corn is high (9.2 g per kg) [16], corn-driven diets tend to have higher Leu-to-Lys ratios. However, especially in Western Europe, wheat-based diets are more commonly used and because wheat is rather low in total Leu (7.2 g per kg) [16], formulating wheat-based diets with an adequate Leu content is more challenging. As the performance in the highest Leu-to-Lys ratio of 1.20 and 1.25 reached a plateau, it seems not likely that the higher optimal ratios can be found in wheat-driven diets.

In rat models, Leu can enhance the muscle protein synthesis via the modulation of the mTOR pathway [29,30]. A comparable upregulation of mTOR due to dietary Leu supplementation also exists in broilers [12,31]. A high dietary Leu, Val and Ile in broilers causes a higher mRNA expression of eukaryotic translation elongation factor 2 affecting the elongation process in protein synthesis in the breast muscle (*Pectoralis major*) [12]. Thus, the BCAAs, in particular Leu, are important for maximizing protein accretion in meat. Kriseldi et al. [20] examined the interaction among BCAAs in a full factorial central composite design. The breast meat yield was maximized with the highest SID Leu-to-Lys ratio (1.90); however, less meat yield was gained with high SID Val-to-Lys or SID Ile-to-Lys ratios. On the contrary, diets exceeding the broiler breeders’ recommendations for Leu by 35% or 60% neither impact the protein synthesis pathway (mTOR) nor the protein degradation pathway (eukaryotic translation initiation factor 2A) nor muscle growth [32]. The lack of response to high Leu (up to 60% higher than the known broiler requirements) suggests that the optimal Leu-to-Lys ratio for muscle growth was met and the protein synthesis was already maximized. Unfortunately, carcass data did not allow for an estimation of the recommended SID Leu-to-Lys ratio (Table 4). A mean SID Leu-to-Lys ratio of 1.03 [24] and 1.04 [25] are recommended for broilers being limited to earlier growing birds and consequently, the recommendations values in this study provide a basis for a proper Leu feeding to the finisher broilers.

Ullrich et al. [8] formulated wheat-based diets with a crude protein content of 21.5%, 20.5% and 20.0% in the starter, grower, and finisher period, respectively. The CP content was then lowered in steps of 1%-point in each phase, leading to finisher diets with CP levels of 20%, 19%, 18% and 17%, respectively. During the finishing phase in the last week of the fattening period (week 5), they noted that the nitrogen content in the excreta was significantly lower, when animals were fed with the lowest CP level in comparison to the control diet (31.7 g/kg dry matter vs. 50.0 g/kg dry matter) [8]. At the same time, the nitrogen-retention efficiency significantly increased from 61.9% (control) to 71.5% in the 17% CP diet [8], which is a comparable CP level as in the current study (16.7%). It can be assumed that at least in the treatments with a sufficient Leu-to-Lys ratio, the nitrogen excretion will be low in comparison to the industry standard, even though we cannot evaluate this hypothesis because no further environmental parameters were measured in this study.

## 5. Conclusions

In finisher broilers fed with a wheat-based diet, Leu deficiency is associated with the limitation of growth and a reduction in carcass yield. Leu supplementation alleviates the loss growth and carcass yield. To achieve 95% and 98% of the maximum response for BWG, FI and the EEF, 0.96 and 1.025 of SID Leu-to-Lys ratio are needed in finisher broiler chickens, respectively. These values are lower than the breeders’ recommendations, but in good agreement with recent research. It can be assumed that the optimal rearing condition during the trial contributes to comparable low SID Leu-to-Lys estimates.

## Figures and Tables

**Table 1:** Feed ingredients and chemical composition [g/kg] of the common starter (day 0–10) and grower (day 10–19) feeds and the basal finisher diet (day 19–33) with a deficient standardized ileal digestibility (SID) leucine (Leu) to SID lysine (Lys) ratio of 0.9. Leu has been added on top to the experimental diets in steps of 0.05 to achieve the eight treatments varying in the SID Leu-to-Lys content between 0.9 and 1.25.

Ingredients [g/kg Diet]	Starter Diet (d 0–10)	Grower Diet (d 10–19)	Basal Finisher Diets (d 19–33), SID Leu-to-Lys 0.90
Wheat	457.70	486.35	575.76
Triticale	150.00	150.00	200.00
Soybean meal (48%)	275.00	230.00	70.00
Sunflower meal	30.00	40.00	50.00
Corn starch			10.00
L-Glutamic acid			5.00
Soy oil	43.00	52.80	37.60
Premix ^1^	5.00	5.00	5.00
Limestone fine	13.30	11.00	11.30
MCP	6.00	3.00	2.40
Salt	1.70	1.60	0.90
NaHCO_3_	3.10	3.00	3.60
K_2_CO_3_	-	-	4.60
L-Lysine HCl 79%	4.10	4.10	6.22
L-Methionine 99%	3.65	3.25	2.77
L-Threonine 99%	2.45	2.05	2.54
L-Tryptophan 98%	-	-	0.13
L-Valine 96.5%	1.50	1.35	2.31
L-Arginine 98.5%	2.15	2.05	3.77
L-Isoleucine 90%	1.00	0.95	1.96
L-Histidine HCl-H_2_O 72%	0.30	0.25	0.66
Glycine 98.5%	-	3.20	1.87
L-Phenylalanine 99%	-	-	1.46
Phytase (1000 FTU, Danisco Animal Nutrition)	0.05	0.05	0.05
Ronozyme^®^ Multigrain ^2^			0.10
Calculated ingredients [g/kg]			
AME_N_, MJ/kg	12.55	12.97	12.97
Crude protein	225.1	212.8	166.8
SID Lys	12.51	11.50	9.45
SID Leu	13.56	12.48	8.50
SID Leu:Lys	1.08	1.08	0.90
Ca	9.0	7.5	7.1
Available phosphorus	4.6	3.9	3.6

^1.^ Provides per kg feed: vitamin A (Retinyl acetat) 10,000 UI; vitamin D_3_ (cholecalciferol) 2500 UI; vitamin E (alpha-tocopherol) 50 mg; vitamin K_3_ (menadione) 2.3 mg; vitamin B_1_ (thiamin) 2.0 mg; vitamin B_2 (_riboflavin) 7.5 mg; vitamin B_6_ (pyridoxine-HCl) 3.5 mg; vitamin B_12_ (cyanocobalamin) 20 µg; Niacin 35 mg; D-pantothenic acid 12 mg; Choline chloride 460 mg; Folic acid 1.0 mg; Biotin 0.2 mg; Fe (from FeSO_4_·H_2_O) 80 mg; I (from KI) 1.2 mg; Cu (from CuSO_4_·5H_2_O) 12 mg; Mn (from MnO) 85 mg; Zn (from ZnSO_4_ H_2_O) 60 mg; Se (E-8) (from Na_2_SeO_3_) 0.25 mg. ^2^ Ronozyme^®^ is a combination of xylanase, beta-glucanase, cellulase and xyloglucanase enzymes.

**Table 2:** Analyzed total amino acid and crude nutrient composition of the starter (day 0–10), grower (10–19) and experimental finisher diets (day 19–33) containing eight graded ratios of standardized ileal digestible (SID) leucine (Leu) to SID Lysine (Lys).

Total Amino Acid Content [%]	Starter Diet	GrowerDiet	Finisher Diet(*0.90*)	Finisher Diet (*0.95*)	Finisher Diet (*1.00*)	Finisher Diet (*1.05*)	Finisher Diet (*1.10*)	Finisher Diet (*1.15*)	Finisher Diet (*1.20*)	Finisher Diet (*1.25*)
Arginine	1.55	1.48	1.14	1.14	1.13	1.14	1.13	1.14	1.15	1.16
Histidine	0.54	0.52	0.36	0.37	0.37	0.37	0.37	0.38	0.38	0.38
Isoleucine	0.97	0.93	0.67	0.71	0.70	0.70	0.71	0.70	0.69	0.69
Leucine	1.53	1.49	0.91	0.97	1.02	1.07	1.11	1.18	1.19	1.25
Lysine	1.37	1.28	1.02	1.03	1.00	1.00	0.99	0.98	1.03	1.01
Methionine	0.63	0.56	0.48	0.48	0.48	0.46	0.49	0.46	0.47	0.46
Cysteine	0.33	0.35	0.26	0.26	0.26	0.27	0.26	0.27	0.26	0.27
Threonine	0.96	0.89	0.67	0.68	0.67	0.67	0.67	0.67	0.68	0.67
Phenylalanine	1.04	0.98	0.76	0.77	0.77	0.77	0.77	0.78	0.77	0.79
Valine	1.12	1.06	0.82	0.84	0.84	0.85	0.85	0.85	0.84	0.86
Proximate [%]										
Crude protein	22.8	21.7	16.7	16.5	16.8	16.8	16.7	16.8	16.8	16.9
Crude fat	6.0	6.9	5.3	5.5	5.3	5.3	5.4	5.4	5.3	5.4
Crude ash	5.2	4.7	4.2	4.3	4.0	4.1	4.1	4.1	4.2	4.2
Crude fiber	2.7	2.9	2.9	2.8	2.9	2.9	2.9	3.2	2.9	3.0

Italic values in brackets refer to the calculated SID Leu to SID Lys ratio; SID = standardized ileal digestible.

**Table 3:** Performance parameters of finisher birds (day 19 to 33) fed with different ratios of standardized ileal digestible (SID) leucine (Leu) to SID lysine (Lys).

Treatment (SID Leu to Lys)	Body Weight, Day 33 [g]	Body Weight Gain, Day 19–33 [g/d]	Average Daily Feed Intake, Day 19–33 [g/d]	FCR [g/g]	European Efficacy Factor
1 (0.90)	2290 ^a^	92 ^a^	148 ^a^	1.609 ^ab^	565 ^a^
2 (0.95)	2369 ^ab^	98 ^ab^	157 ^ab^	1.612 ^ab^	597 ^ab^
3 (1.00)	2439 ^bc^	102 ^bc^	164 ^bc^	1.612 ^ab^	617 ^abc^
4 (1.05)	2475 ^bc^	104 ^bc^	166 ^bc^	1.600 ^ab^	643 ^bc^
5 (1.10)	2453 ^bc^	103 ^bc^	166 ^bc^	1.605 ^ab^	637 ^bc^
6 (1.15)	2458 ^bc^	103 ^bc^	167 ^bc^	1.612 ^ab^	627 ^abc^
7 (1.20)	2502 ^c^	107 ^c^	168 ^bc^	1.572 ^b^	671 ^c^
8 (1.25)	2456 ^bc^	102 ^bc^	168 ^c^	1.642 ^a^	611 ^ab^
SEM	27	1.5	2.4	0.0134	15

^a,b,c^ Values in the same column with different letters are significantly different (*p* < 0.05). Differences between treatments were assessed by Tukey’s test to account for multiple comparisons.

**Table 4:** Carcass parameters of birds at day 34.

Treatment (SID Leu-to-Lys)	Carcass Yield [g]	Dressing [%]	Breast Meat Yield [g]	Thighs Yield [g]	Breast Weight Relative to Carcass Weight [%]	Thighs Yield Relative to Carcass Weight [%]
1 (0.90)	1651 ^a^	72.2 ^a^	412 ^a^	505 ^a^	24.8 ^a^	30.6 ^b^
2 (0.95)	1750 ^b^	73.2 ^ab^	445 ^ab^	527 ^ab^	25.3 ^ab^	30.2 ^b^
3 (1.00)	1775 ^bc^	72.4 ^ab^	473 ^c^	529 ^ab^	26.6 ^bc^	29.8 ^ab^
4 (1.05)	1810 ^bc^	73.1 ^ab^	486 ^b^	525 ^ab^	26.8 ^bc^	29.0 ^a^
5 (1.10)	1784 ^bc^	72.5 ^ab^	465 ^bc^	522 ^ab^	26.0 ^abc^	29.3 ^a^
6 (1.15)	1816 ^bc^	73.8 ^b^	503 ^c^	534 ^b^	27.7 ^c^	29.4 ^ab^
7 (1.20)	1833 ^c^	73.0 ^ab^	493 ^c^	541 ^b^	26.9 ^bc^	29.6 ^ab^
8 (1.25)	1803 ^bc^	73.3 ^ab^	486 ^c^	522 ^ab^	26.9 ^c^	29.0 ^a^
SEM	16	0.31	8	6	0.33	0.22

^a,b,c^ Values in the same column with different letters are significantly different (*p* < 0.05). Differences between treatments were assessed by Tukey’s test to account for multiple comparisons. SID = standardized ileal digestible.

**Table 5:** Estimation of nutritionally required standardized ileal digestible (SID) Leu-to-Lys ratio to achieve 95% and 98% for maximum performance.

	95% of Max Performance			98% of Max Performance		
	Estimate	LCL *	UCL **	Estimate	LCL *	UCL **
Final body weight (BW), g						
Plateau	2476	2444	2509			
SID Leu to Lys	0.930	0.900	0.960	0.994	0.924	1.063
BW gain, g per d						
Plateau	104.5	102.7	106.2			
SID Leu to Lys	0.962	0.923	1.001	1.025	0.951	1.098
Average daily feed intake, g/d						
Plateau	167.8	165.0	170.7			
SID Leu to Lys	0.960	0.920	1.000	1.023	0.947	1.099
European Efficacy Factor						
Plateau	639	621	658			
SID Leu to Lys	0.960	0.895	1.025	1.023	0.901	1.146

* LCL = lower 95% confidence limit; ** UCL = upper 95% confidence limit.

## Data Availability

The data presented in this study are not publicly available due to privacy restrictions.
